# Judging time-to-passage of looming sounds: Evidence for the use of distance-based information

**DOI:** 10.1371/journal.pone.0177734

**Published:** 2017-05-22

**Authors:** Rosa Mariana Silva, João Lamas, Carlos César Silva, Yann Coello, Sandra Mouta, Jorge Almeida Santos

**Affiliations:** 1 Center for Computer Graphics, Guimarães, Portugal; 2 Department of Basic Psychology, School of Psychology, University of Minho, Braga, Portugal; 3 Department of Informatics, University of Minho, Braga, Portugal; 4 INESC TEC, Institute for Systems and Computer Engineering Technology and Science, Porto, Portugal; 5 Cognitive and Affective Sciences Laboratory (SCALab), UMR 9193 CNRS, University of Lille, Villeneuve d’Ascq, France; 6 Centro Algoritmi, University of Minho, Guimarães, Portugal; University of Muenster, GERMANY

## Abstract

Perceptual judgments are an essential mechanism for our everyday interaction with other moving agents or events. For instance, estimation of the time remaining before an object contacts or passes us is essential to act upon or to avoid that object. Previous studies have demonstrated that participants use different cues to estimate the time to contact or the time to passage of approaching visual stimuli. Despite the considerable number of studies on the judgment of approaching auditory stimuli, not much is known about the cues that guide listeners’ performance in an auditory Time-to-Passage (TTP) task. The present study evaluates how accurately participants judge approaching white-noise stimuli in a TTP task that included variable occlusion periods (portion of the presentation time where the stimulus is not audible). Results showed that participants were able to accurately estimate TTP and their performance, in general, was weakly affected by occlusion periods. Moreover, we looked into the psychoacoustic variables provided by the stimuli and analysed how binaural cues related with the performance obtained in the psychophysical task. The binaural temporal difference seems to be the psychoacoustic cue guiding participants’ performance for lower amounts of occlusion, while the binaural loudness difference seems to be the cue guiding performance for higher amounts of occlusion. These results allowed us to explain the perceptual strategies used by participants in a TTP task (maintaining accuracy by shifting the informative cue for TTP estimation), and to demonstrate that the psychoacoustic cue guiding listeners’ performance changes according to the occlusion period.

## Introduction

Temporal estimations underlie many functional interactions with moving objects. For instance, when perceiving cars getting closer on a busy road, we usually perform estimations of how long it would take the vehicles to reach our location, and we take this into account to decide whether it would be safe to cross the road [1]. In many scenarios, estimating the remaining time for moving objects to reach our own position is of crucial importance for safe navigation or for successful interactions with objects in our surroundings. Accurately perceiving the dynamic cues from approaching objects might facilitate successful interactions. Nevertheless, there are situations in which these dynamic cues may not be available until the moment of contact or passage, such as when the moving object is occluded by other objects or the sound of the object moving is masked during its approaching trajectory. There is an extensive body of literature devoted to the study of dynamic events that specifically focuses on how humans perceive approaching objects and on how trajectory cues (either visual, acoustic, or both) can provide information for the perceptual judgment of approaching events (also referred to as looming events). However, such studies are predominantly centred the monocular and binocular cues informative for visual looming events [2–9; 10, 11], while fewer studies have been conducted on the psychoacoustic cues used in auditory looming events [12,13].

These studies have traditionally addressed two main questions: (1) how accurately can the perceptual system provide temporal estimations of moving objects and (2) which cues guide the perceptual judgement of looming events.

### Accuracy in perceptual judgments for looming events

Regarding the first line of inquiry–the accuracy in perceptual judgments for looming events–studies on the visual modality have reported that when asked to estimate the time to contact (TTC) of an approaching object on a collision trajectory, participants consistently judge the stimulus as having contacted them sooner than it physically would [2, 14, 15]. This anticipatory effect has been reported in studies where participants judge the time to arrival (TTA) [16], or the time to passage (TTP) [7] of a visual looming event. Schiff and Oldak (1990) tested vehicles in an approaching trajectory and asked participants to press a button when they estimated that the automobile would reach them [17]. One important detail of this study was that the visual stimulus would disappear during the final part of the trajectory. This period was termed “occlusion period” and participants were asked to take into consideration that, while not visible, the vehicle would keep approaching under the same motion conditions (speed and trajectory). Results indicated that anticipations of the TTA were between 20% and 40% of the actual TTA.

Similarly, previous studies have also revealed anticipatory effects in perceptual judgments involving looming auditory stimuli. The first evidence came from a particular condition in the study of Schiff & Oldak (1990) where participants judged the TTA of the approaching vehicle’s sound. Results showed that, when compared with performance in the visual and audiovisual conditions, participants were less accurate in estimating the TTA. For the auditory condition, results showed a greater anticipatory effect, i.e. the vehicle was perceived to arrive sooner than it actually did. Moreover, Rosenblum, Wuestefeld and Saldaña (1993) also tested the effect of stimulus duration and the amount of occlusion in the accuracy of a TTP judgment. When judging the passage of a sound recorded from an approaching car, participants overall anticipated passage in 84% of the trials (judged the car to pass sooner than they physically did). Moreover, the accuracy declined as the amount of occlusion increased.

Other studies in the auditory modality used loudness change tasks, where participants were asked to provide loudness judgments on pairs of sounds with varying intensity by saying how much one sound changed in loudness in comparison with the other. A pair of sounds was composed by a stimulus increasing in intensity and another decreasing in intensity. Participants were instructed to answer if stimuli changed equally or one of the stimuli changed more in loudness than the other. These studies assume that sounds increasing in intensity are perceived as a sound source approaching the listener. Therefore, judgements of loudness change between a pair of sounds with varying intensity could provide information regarding dynamic events. Neuhoff [18–20] conducted several experiments to study whether loudness magnitude was equally perceived regardless of the direction of change. Using a sinusoid tone as a stimulus, his major finding was that when individuals were asked to evaluate the amount of loudness change in a pair of sounds with increasing and decreasing intensity, the former was evaluated as changing more than the latter, despite their equal amount of intensity variation. In other words, participants judged sounds increasing in intensity as changing more in loudness than those decreasing in intensity. Interestingly, the spectral features of the auditory source seem to play a crucial role in the persistence of this effect. Neuhoff (1998) compared sinusoid tones with complex tones and white-noise, and found that the previous bias did not occur when white-noise was used as the auditory stimulus [18]. The lack of evidence for this increasing intensity phenomenon when using white-noise was replicated in further studies [21–23].

The perceptual bias when judging looming events has not been interpreted as an error of the subject but as an adaptive response, to ensure a safety time margin to act [20]. It may be the case that the auditory system might simply not be able to provide accurate temporal estimates. However, it is possible that this specificity of the auditory system works to improve humans’ survival chances by functioning as a warning system that prepares and gives the listener time to act, as well as increasing this time span in which the person must act [24].

### Perceptual cues guiding the judgement of looming events

When inquiring about the cues guiding the perceptual judgement of looming events, most studies have been focused on the visual modality. These studies have shown that participants demonstrate a higher anticipatory effect when judging TTC of visual stimuli under monocular, relative to binocular, viewing conditions [10]. Additionally, changing disparity cues seems to reduce the anticipatory effect in small objects (smaller than an angular size of 0.5° as reported by Heuer, 1993a [11]; smaller than angular size of 0.7° as reported by Gray & Regan, 1998 [10]). When both looming and binocular cues are available, the magnitude of the anticipatory effect in TTC judgment decreases to values between 1.3 and 2.7% of the actual TTC [10]. In sum, TTC studies in the visual modality, show that participants can use different cues, alone or combined, to obtain improved performance. The use of one or more cues is generally associated with the specificities of the task, such as the trajectory of the looming event, the size of the object, and/or the amount of information available to provide an estimate.

In an analogous way to what happens in the visual modality, some studies addressed the study of acoustic and psychoacoustic cues that guide the judgement of looming events. Monaural cues, such as intensity growth, and binaural cues, such as interaural time difference (ITD) and interaural level difference (ILD), are important for an accurate perception/identification of the sound source in space (Middlebrooks & Green, 1991)[25]. Therefore, to better understand the mechanisms that guide the timing estimation, all cues should reliably match their spatiotemporal configuration. In a TTP study of Rosenblum, Carello & Pastore (1987) an ambulance siren was used as auditory stimulus [12]. These authors tested the weight of three acoustic variables in timing estimates, while instructing participants to press a button when they perceived the ambulance crossing their sagittal plane. The timing estimate was tested in a series of conditions, where ITD, Doppler effect, and intensity change could be kept either congruent or incongruent with each other. Congruency happened when the moment of passage of the stimulus coincided for all the variables. The key result from this study was that intensity change was the most dominant cue, followed by ITD and then Doppler. The highest accuracy on the judgment of passage was obtained when participants relied on intensity change. This result was also replicated by Bach, Neuhoff, Perrig and Seifritz (2009), where participants were asked to rate loudness change [13]. However, it is important to note that the sound pressure level of full motion cues stimuli was lower than the intensity only stimuli. Also, in both studies the trajectory of the stimulus was parallel to the interaural axis, and not presented sagittally to it, which is extremely relevant for studies aiming to highlight the perceptual mechanisms underlying interactions with objects or collision avoidance strategies.

In this paper, we focused on the ability of subjects to perform temporal judgments in a TTP task with approaching sounds. Despite the fact that the studies above have endorsed many aspects of events in approaching trajectories, such the type of stimuli in timing estimation tasks [17] and the role of the occlusion period along the trajectory [1], key questions still remain to be addressed. Namely, what are the different psychoacoustic cues that predict performance in a TTP task with a sound source coming towards the listener, and what is the impact of each one of these cues on performance. Therefore, the aim of the present study was to understand how listeners perceive the passage of looming sounds. Specifically, our first goal was to evaluate whether participants are able to accurately judge the passage of an approaching binaural white-noise sound source. Considering the studies mentioned above on auditory TTA [17] and TTP [1], which presented stimuli with an occlusion period, we would expect that participants would anticipate TTP. However, as the studies on loudness change conducted by Neuhoff (1998) [18], Seifritz *et al*. (2002) [21] and Ghazanfar *et al*. (2002) [22] have shown that white-noise stimuli are judged with greater accuracy in comparison with harmonic tones, we hypothesize that participants would be able to accurately estimate time to passage, despite the occlusion period. As far as we know, no study has yet tested the role of the occlusion period on the anticipation of looming sounds in the sagittal plane. Therefore, it is also the goal of this study to test the effect of the occlusion period on the temporal estimation of looming white-noise sounds. Secondly, we were interested in evaluating the most relevant variables contributing to the estimation of passage of looming sounds. As such, we analysed several physical and psychoacoustic variables, such as sound position, velocity, TTP, and binaural cues (ITD and ILD) in relation with participant’s performance, in order to understand which cues participants rely on the most when judging the TTP of auditory stimuli.

## Method

### Ethics statement

This study was approved by the Ethical Committee of Sciences for Life and Health of the University of Minho (SECVS 031/2015), in Portugal. The experiment was conducted in accordance with the principles stated in the 1964 Declaration of Helsinki. All participants provided written informed consent after having been debriefed on the experimental procedure, safety precautions, anonymity of data collected, and the participants’ right to withdraw from the experiment at any time.

### Participants

Six participants (four males and two females, one of whom left-handed) with ages ranging from 24 to 33 years old (Mean = 27.17, SD = 3.13) took part in the experiment after having participated in a pilot study of auditory localization. All subjects had normal hearing as confirmed with an audiogram test on a Beltone 109 audiometer.

### Stimuli and materials

Experiments were conducted in a darkened room at the Laboratory of Visualization and Perception in the University of Minho. The stimuli consisted of binaural white-noise (frequency rate = 44.1 KHz; intensity magnitude from 77 to 89 dB) processed in free-field and generated in Matlab® using the MIT HRTF (head related transfer function) database [26]. The anechoic sound was divided in several portions (from a minimum of 10 samples per second to a maximum of 17, depending on the stimulus’ travelled distance) along the trajectory and each small sample was convolved with the corresponding HRIR (head related impulse response)–the time domain representation of the HRTF—for each position, thus originating a dynamic sound. This convolution of the HRIR with white-noise maintains the ITD and ILD temporally updated in order to correspond to a new spatial location along the trajectory [27]. Stimuli were generated at 0° elevation, allowing the observer to perceive the sound at their ear level (defined by the sound projection’s configuration) and disposed in the sagittal plane 1m from participants’ right shoulder. Stimuli were processed by a Realtec Intel 8280 IBA sound card and presented through a set of Etymotic ER-4P MicroPro in-ear headphones.

Forty-nine looming sounds with the duration of 1 second were generated in Matlab®. As in previous studies on visual TTP [7], seven simulated initial distances– 0.78, 0.85, 0.93, 1, 1.09, 1.18, 1.29 m–were combined with seven simulated constant velocities– 0.76, 0.83, 0.91, 1, 1.1, 1.2, 1.32 m/s–resulting in 49 total theoretical TTP (TTPt): 24 that passed the subject before 1 s (passage by the participant occurred), one that stopped at the ear level precisely at 1 s, and 24 that would pass after 1 s (passage by participant did not occur).

Stimuli were blocked according to the amount of occlusion. All trials lasted for 1 s and differed only in the amount of occlusion, and consequently the offset of the stimulus presentation. The minimum occlusion was 0.1 s when stimulus presentation was 0.9 s (10% occlusion), the intermediate occlusion time was 0.3 s when stimulus presentation was 0.7 s (30% occlusion), and the maximum occlusion time was 0.5 s when the stimulus had been presented for 0.5 s (50% occlusion). Considering the different occlusion conditions, final Time-to-Passage (TTPf) was computed as the ratio between final distance (fDist) and speed. Moreover, variables have different range of values and Points of Objective Simultaneity (POS) (*see* S1 Table). POS is defined as the moment where the stimulus is precisely aligned with the ear plane of the participants. This experiment consisted of a total of 147 stimuli (49 total TTPt x 3 occlusion levels).

### Procedure

A control study was conducted to evaluate whether participants could accurately discriminate discrete bursts of white-noise at different positions of the looming trajectory. All the participants in this study were able to perform the task under the mean error values reported in the literature for sound localization experiments, with average azimuth errors ranging between 6.71˚ and 14.86˚ at 0˚ elevation [28].

Before the experimental session, participants were exposed to six stimuli (3 that would pass by the participant and three that would not pass be the participant) in order to evaluate if they understood the experimental task.

During the experimental sessions participants were seated in a darkened room and a chin rest was used to stabilize and minimize head movements. Participants were told that they would hear sounds approaching them, at a trajectory 1 m from their right shoulder, similarly to the study of Mouta *et al*. (2012). They were instructed to estimate the time to passage of the auditory stimuli using the plane defined by their ears (perpendicular to the sagittal plane) as a reference for the point of passage. They were also told that stimuli would not be completely presented. At a certain portion of presentation time, stimuli would be interrupted and would remain inaudible until the end of the trial (occlusion period). During this period, participants were instructed to estimate when the sound, approaching at the same speed and along the same trajectory, would pass by their ear plane. By the end of the trial an auditory beep was presented, as a temporal marker, signalling the moment when the participant had to provide their estimate of passage. This estimate would be provided by pressing one of two response buttons, depending on whether the stimulus was perceived to pass before or after the beep (2-AFCT). Stimuli presentation was always 1 s, despite different periods of occlusion. The temporal marker was always presented after total stimuli presentation (1s) and had a total duration of 0.75 s. Participants were requested to answer during the temporal marker presentation that served also as the inter-stimulus interval (ISI). The experiment consisted of 20 repetitions of each stimulus divided by 12 blocks (each with 4 stimulus repetitions). Stimuli were pseudo-randomized by block and counterbalanced by participant. This experiment had a total duration of approximately 70 minutes per participant (each block lasted approximately 6 minutes). The temporal marker was empirically validated as a suitable marker by running a pre-test between an auditory marker, a binaural tone (fundamental frequency = 1500 Hz) and a visual marker, a flash, both lasting for 1 s. The binaural beep demonstrated less variability in the results and therefore it was selected as the temporal marker to signal the moment of judgment, thus maintaining the experiment as a purely auditory modality task.

### Psychoacoustic measures

Since our stimuli consisted of a broadband sound source presented in the sagittal plane, we considered that the psychoacoustic cues most relevant for analysis would be the binaural cues of ILD and ITD. In order to extract ILD, stimuli from all conditions were recorded using Brüel & Kjӕr head and torso simulator (HATS) type 4128-C and Pulse Analyzer type 3560-C. These measures were divided into left and right channels using Matlab® 2013b and served as input for the analysis carried out for the extraction of the psychoacoustic variables. To calibrate each channel a stimuli corresponding to the POS for each occlusion condition was used (starting at 1 m from the observer and travelling at 1 m/s). The psychoacoustic measure extracted for each channel, was the Instantaneous Loudness in sones (according to the model of Time-Varying Sounds from Moore, Glasberg & Baer [29]), using Psysound3 Toolbox. Sone is a unit of perceived loudness proposed by Stevens and Davis (1938) [30] where 1 sone is equivalent to a tone of 1 kHz at 40 dB. This measure of loudness enables the experimenter to add or subtract sones without introducing any bias to the data, as the sone unit is proportional to loudness. Instantaneous Loudness was analysed for each millisecond of the stimuli’s duration. A moving average of 50 samples was applied to smooth the loudness curve and subsequently a magnitude difference between an initial period of time (first 50 ms) and a final period of time (last 50 ms) for each sound was calculated. Subsequently, an Interaural Level Difference from Instantaneous Loudness in sones (ILD_Loud_) was calculated as the difference between right and left ear canals from this magnitude difference.

From the original equation proposed in the model of Instantaneous Loudness [29]:

$$N(t)=\int_{nmin}^{nmax}N^{\prime }(n,t)dn$$

where *n* is the equivalent rectangular bandwidth (ERB) number for any given frequency which can be calculated as *n* = 21.4_log_10(0.00437 *fc* + 1), and *t* the integration time (1 ms).

We present instantaneous loudness as *N*_*L*_*(t)* for the left and *N*_*R*_*(t)* for right channels,

$$N_{L}(t)=\int_{nmin}^{nmax}N\prime (n_{l},t){dn}_{l}$$

$$N_{R}(t)=\int_{nmin}^{nmax}N\prime (n_{r},t){dn}_{r}$$

where *n*_*l*_ and n_*r*_ are the equivalent to *n* from the original model equation and *t = 50ms* due to the 50 samples’ moving average. The Loudness magnitude was calculated for each ear canal as,

ΔL=L(t)final−L(t)initial

ΔR=R(t)final−R(t)initial

Finally, ILD_Loud_ was obtained from,

$${ILD}_{Loud}=\Delta L-\Delta R$$

Interaural Temporal Difference (ITD) was calculated from the time differences between channels at the beginning of the stimulus presentation (ITD_initial_) and from the time differences between channels at the final moment of stimulus presentation (ITD_final_). Two methods were used to calculate ITD_initial_ and ITD_final_: empirically (calculated from the distance of the first position of the sound source to each ear by the speed of sound–ITD_initial_ empirical—or from the distance of the last position of the sound source to each ear by the speed of sound–ITD_final_ empirical—in milliseconds) and according to Rayleigh’s model [31]:

$$ITD=\frac{r(\theta +\sin\left(\theta \right))}{c}$$

Where *r* = radius of the head in meters (0.09m); *ϴ* = the angle between the sound source and the median plane of the head in radians; *c* = speed of sound in air (≃343.2 m/s). From this model, the ITD was also calculated for the angle of the first position of the sound source to each ear–ITD_initial_ model–and from the angle of the last position of the sound source to each ear–ITD_final_ model.

### Analysis

Cumulative Gaussian curves were fitted to the pooled data in order to generate distributions of the proportion of trials in which passage of the auditory stimuli was later than 1 s. We estimated two parameters, the point of subjective simultaneity (PSS) and the standard deviation (SD). PSS was defined as the point at which the TTPt value reached the 50% of “later” responses. A PSS larger than 1s (curves shifted to the right) means that the stimulus was perceived as passing earlier than what was simulated, i.e. an anticipation of passage. SD is inversely related to the slope of the function and provides information about the uncertainty underlying the participants’ performance. We estimated both parameters from individual and pooled data for all participants. Parametric bootstrap [32] was run to obtain 95% Confidence Intervals of the two parameters of the cumulative Gaussian functions for each occlusion condition. The goodness of the fit was tested using the Deviance Statistic, which follows a Chi-square distribution with degrees of freedom equal to the number of data points minus the number of parameters on the model. Larger p-values (>0.05) indicate that the model is a good descriptor of the data. Therefore, p-values close to 1 show that the data adjust to the model of best fit. Akaike Information Criterion (AIC) is presented along with Deviance Statistic as criteria for decision of the preferred model. The minimum value of AIC was considered as a good indicator of the model’s best fit. The proportion of responses was plotted as a function of different variables or predictors; and then AIC was used to decide which model better adjusted to the data.

## Results and discussion

**Fig 1:**
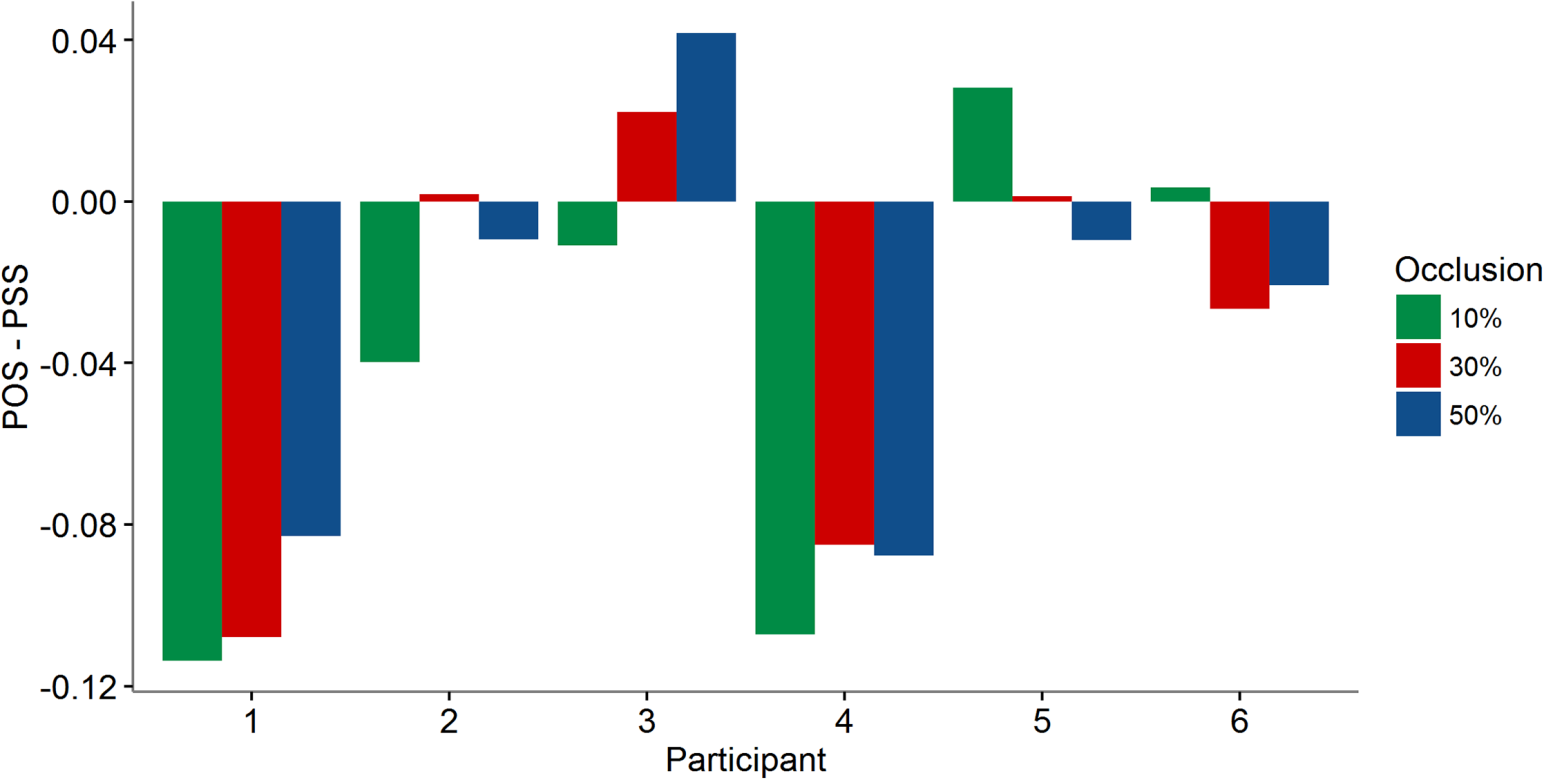
Difference between physical simultaneity (1s) and the PSS (extracted from individual fit as a function of TTPt) plotted for each participant and for the different occlusion conditions.

Accuracy and precision were analysed through the extraction of the parameters PSS and SD from the psychophysical curve of the proportion of “later than beep” responses as a function of TTPt. If participants are accurate we expect a PSS not significantly different from 1 second. On the other hand, if they anticipate the time of the stimuli’s passage, it is expected that the psychophysical curve would shift to the right (PSS > 1). To anticipate the moment of passage means that participants would answer more often that the stimulus has already passed when in fact it has not. Therefore, the curve would be shifted to the right. Regarding precision, the SD parameter will be compared to the visual modality (*see* [7]). PSS and SD parameters were extracted from individual fits. Overall, participants were able to estimate passage regardless of the amount of occlusion. There was no consistent bias on passage estimation among participants. Fig 1 shows the deviation of accuracy from the physical simultaneity (POS = 1) for each participant. We can see that participants 1 and 4 showed a decrease of the PSS as the amount of occlusion increased. On the other hand, participants 2, 3, 5 and 6 showed a greater variability of judgment. We did not find significant effects of the amount of occlusion on accuracy when data is plotted as function of TTPt. After bootstrapping the PSS parameter, we obtained the following values with 95% confidence interval (CI): [1.017, 1.060] for 10% occlusion; [1.011, 1.050] for 30% occlusion; and [1.003, 1.050] for 50% occlusion.

### Physical cues

**Fig 2:**
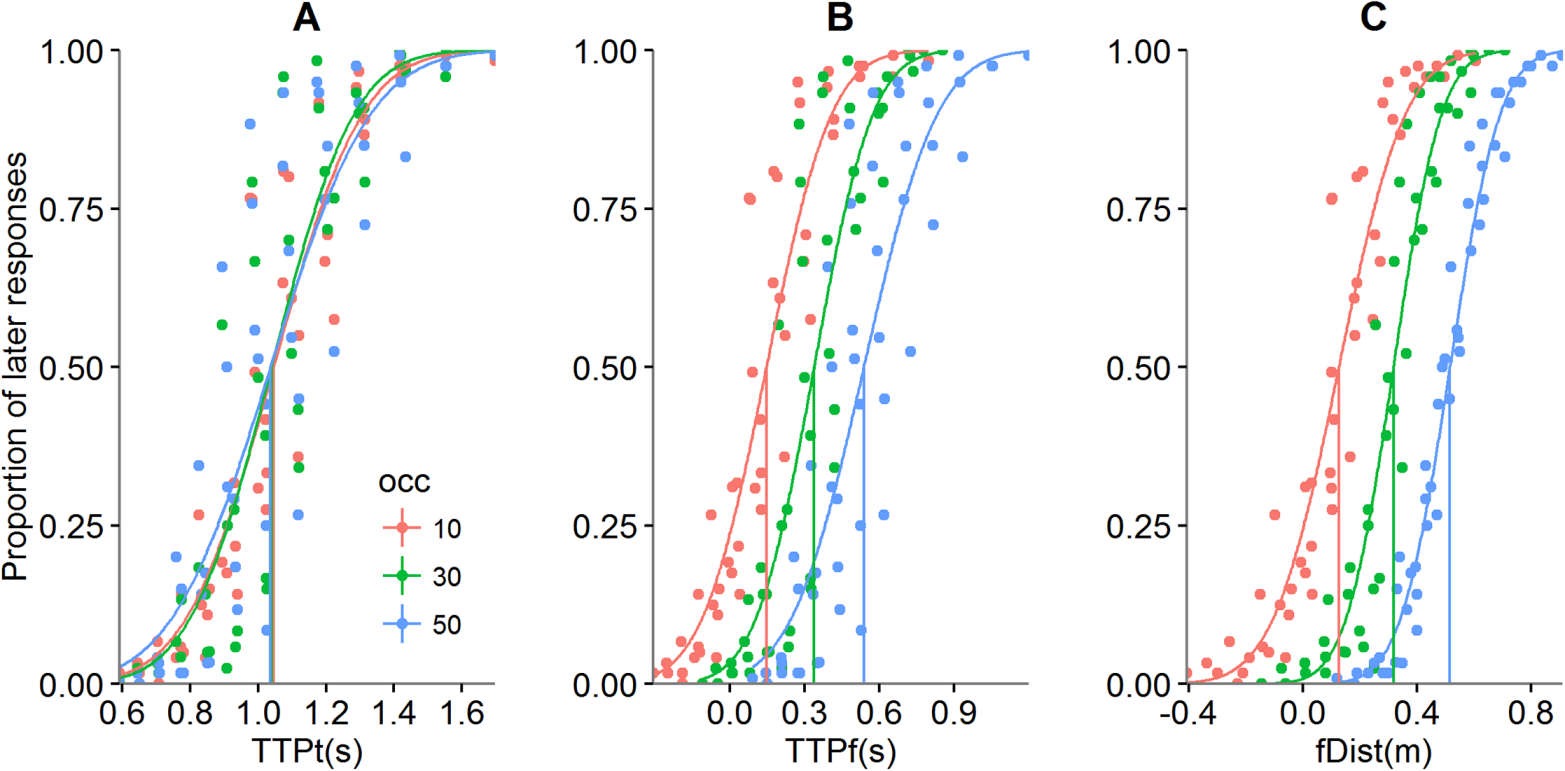
Proportion of “later than beep” responses as a function of TTPt (A), TTPf (B) and fDist (C) for each level of occlusion (red = 10% occlusion; green = 30% occlusion; blue = 50% occlusion). Different PSS are obtained for TTPf and fDist due to the different range of values of the variables (see Method section and Table in S1 Table), and therefore are not due to an effect of occlusion on judgment.

No significant differences were found between participants regarding the accuracy, F(1, 14) = 0.924, *p = 0*.*353*, thus we were able to aggregate data in a pooled distribution. Pooled data regarding the estimation of passage of looming sounds demonstrated no significant differences in the goodness of fit for the three-occlusion conditions. TTPt was equally predictive of the judgment for all occlusions (*see* Fig 2A) as was TTPf (*see* Fig 2B) and fDist (*see* Fig 2C).

**Fig 3:**
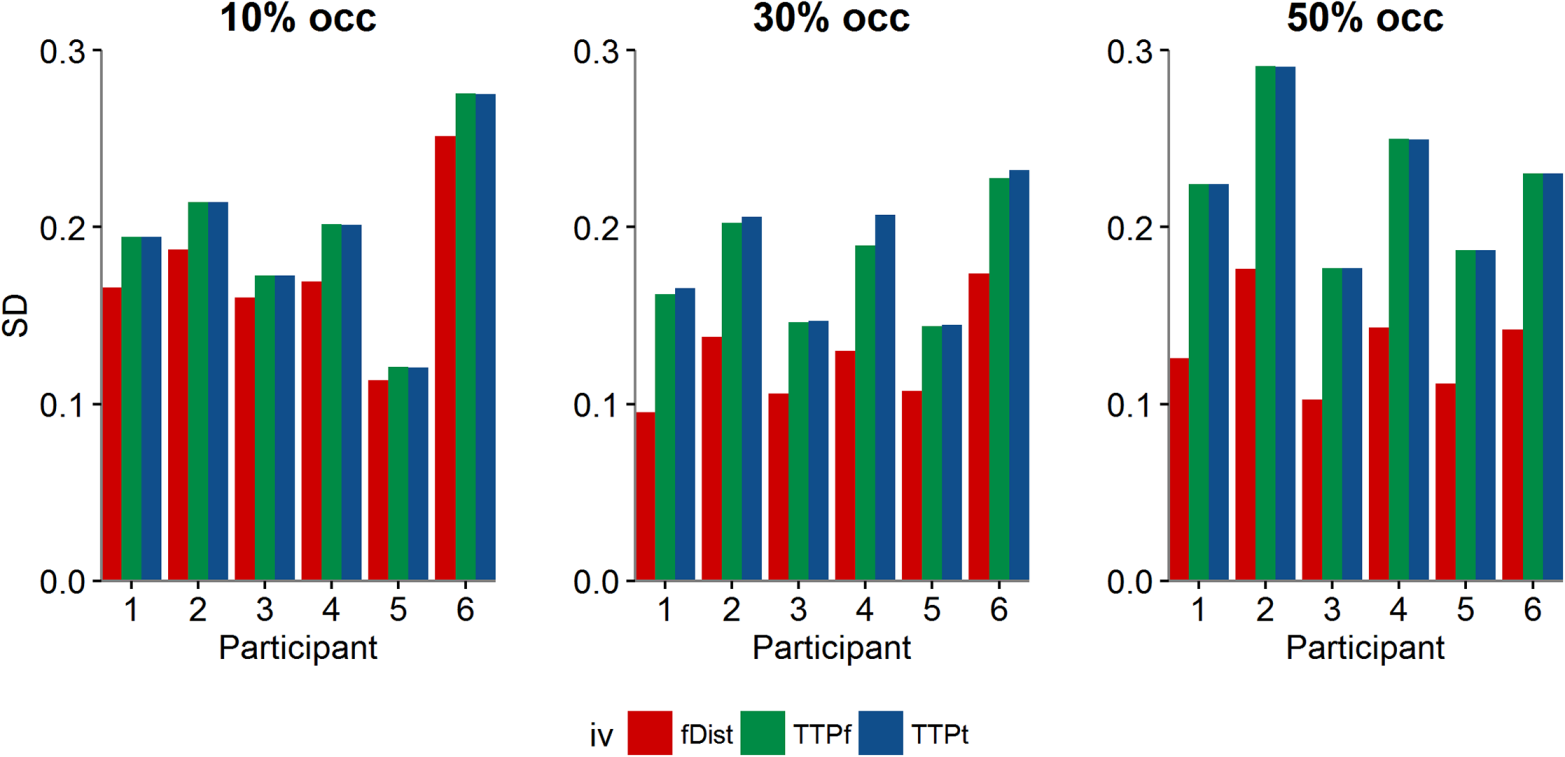
Individual SD values for each participant plotted for each occlusion condition (extracted from fit as a function of TTPt, TTPf and fDist).

Additionally, we plotted the SD extracted from the individual fits as a function of the TTPt for the different occlusions. Significant differences between the occlusions were not found. Bootstrap with 95% CI revealed the following values: [0.173, 0.223] for 10% occlusion; [0.163, 0.216] for 30% occlusion and [0.201, 0.262] for 50% of occlusion. By looking at each of the SD values individually (*see* Fig 3), we found greater SD values for TTPf as the amount of occlusion increases, whereas the uncertainty of the judgment appears to decrease for higher occlusions when data is plotted as function of fDist (smaller SD values). This tendency is constant among participants. An effect of the amount of occlusion on SD was found as a function of fDist. Bootstrap revealed the following values with 95% CI: [0.148, 0.196] for 10% of occlusion, [0.097, 0.131] for 30% occlusion and [0.116, 0.144] for 50% occlusion.

Overall, performance did not deteriorate with the amount of occlusion. Although we could not find a consistent trend in terms of accuracy for all participants, the precision results were very consistent among them. We stress that SD values for fDist were lower when stimuli were presented with 30% and 50% of occlusion in comparison with SD extracted from the fit of TTPt and TTPf for the same occlusion conditions. This indicates that participants seem to rely more on final distance as an informative cue at these levels of occlusion. Therefore, accuracy did not decrease with the increase of uncertainty, because participants relied on different cues to overcome the stimuli’s deterioration. Additionally, as shown in Fig 3, the SD for TTPt and TTPf increases with the amount of occlusion, whereas the opposite is true for fDist. This might indicate that participants change their strategy (the usage of the informative variable) as the amount of occlusion varies.

### Psychoacoustic cues

Psychoacoustic variables were plotted as a function of the proportion of “later than beep” responses for all occlusion conditions. For each variable, the data was fitted with a maximum-likelihood estimation method [32] and the goodness of the fit was assessed through Deviance Statistic and AIC from Generalized Linear Models (GLM) analysis. For the pooled data, the quality of the fit was not different between initial Interaural Time Difference (ITD_initial_) empirical and ITD_initial_ model (*see* Table 1). However, data did not adjust to the psychometric curve and therefore, it was not possible to obtain individual fits for ITD_initial_ empirical regarding all the occlusion conditions.

**Table 1:** Psychoacoustic variables analysed as a function of the proportion of later than beep responses. For each psychoacoustic variable and occlusion period the quality of the fit is presented through Deviance statistic, its' significance value, and AIC.

Pooled Data		Deviance	p	AIC
**ITD**_**initial**_ **empirical**	10% occ	11,998	*p = 1*	42,869
	30% occ	10,698		33,738
	50% occ	5,8339		34,193
**ITD**_**initial**_ **model**	10% occ	12,045	*p = 1*	42,907
	30% occ	10,743		33,789
	50% occ	5,8311		34,172
**ITD**_**final**_ **empirical**	10% occ	19,653	*p = 1*	54,46
	30% occ	4,3236		26,65
	50% occ	1,4062		25,924
**ITD**_**final**_ **model**	10% occ	19,763	*p = 1*	52,67
	30% occ	3,5602		26,236
	50% occ	1,1641		25,783
**ILD**_**Loud**_	10% occ	5,8659	*p = 1*	27,89
	30% occ	18,664	*p = 1*	47,546
	50% occ	x	x	x

**Fig 4:**
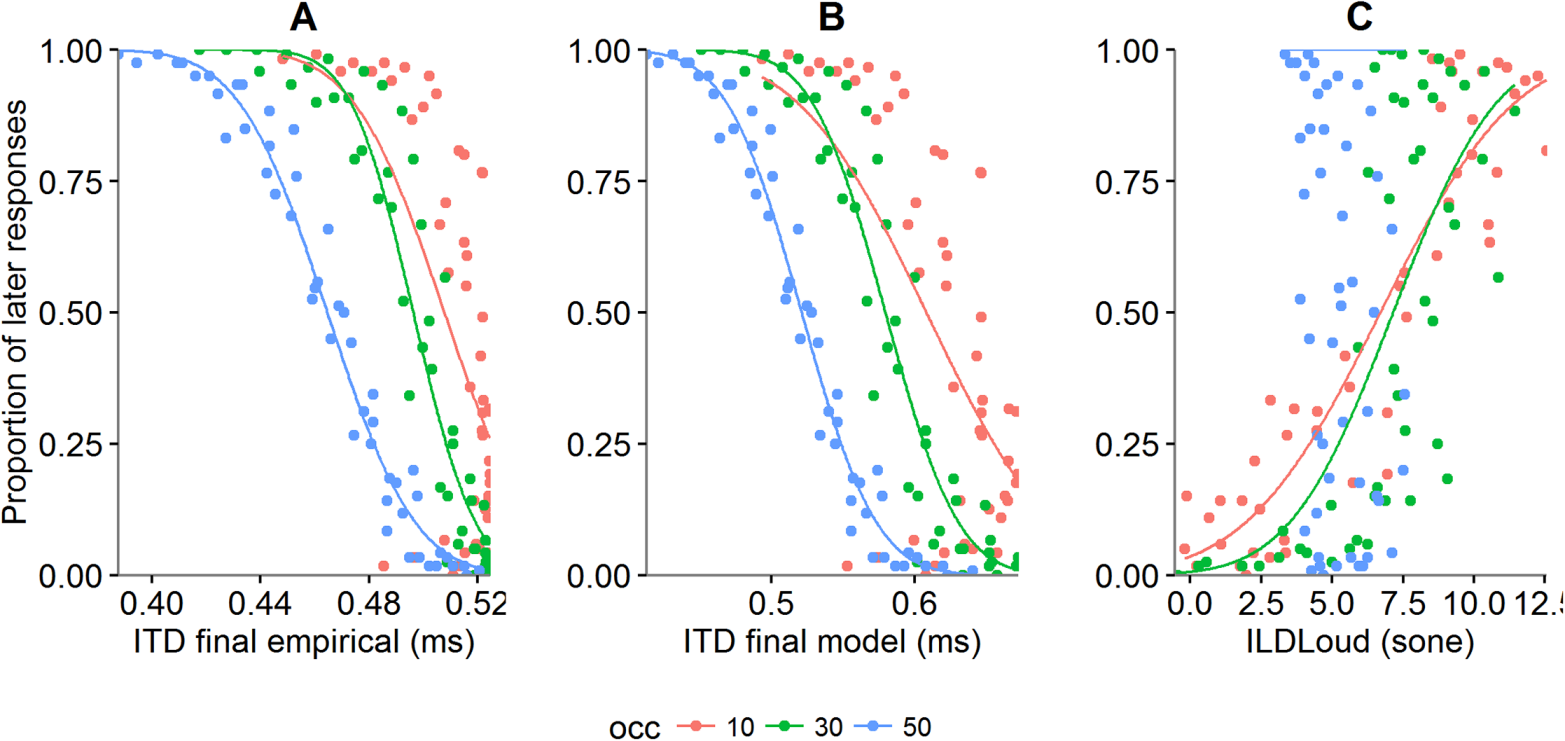
Psychoacoustic variables plotted for each occlusion condition. ITD_final_ empirical (A) ITD_final_ model (B) and ILD_Loud_ (C) are plotted as a function of the proportion of "later than beep" responses.

Deviance Statistic and AIC values do not differ between final ITD (ITD_final_) empirical and model. The goodness of fit is higher for 30 and 50% of occlusion in both variables (*see* Fig 4A and 4B). Moreover, data did not fit for 50% of occlusion for ILD_Loud_ (*see* Fig 4C). Opposite to ITD_final_, ILD_Loud_ best fit was obtained for the 10% occlusion condition. These results highlight the main findings regarding the variables informative for the estimation of stimuli’s passage. It seems that ITD_final_ and ILD_Loud_ have opposite tendencies as a function of the amount of occlusion. As ITD_final_ explains the better performance for 30 and 50% occlusion, ILD_Loud_ appears to be more representative for the performance obtained at 10% occlusion. The switch of quality of fit as a function of amount of occlusion bears resemblance with the change between cues that maintained the performance in the task. More specifically, the drop in the quality of the fit of ITD_final_ as the amount of occlusion decreases could indicate the usage of TTPt as a more reliable cue, since the latter revealed lower SD values for greater amounts of occlusion. Interestingly, the higher values of goodness of fit obtained for 50% occlusion regarding ILD_Loud_ seem to represent the lower uncertainty obtained for final distance as a function of the amount of occlusion.

## General discussion

Our study stemmed from the need to understand how accurate and precise participants were in estimating the passage of approaching sounds. The results indicate that participants readily extract information, including binaural cues, from an auditory looming event. We showed that participants were able to judge passage of looming sounds (white-noise), even when portions of these looming events are occluded. Surprisingly, participants were able to maintain the level of performance despite the duration of stimulus occlusion.

An anticipatory tendency for passage was not found, as previous experiments on auditory TTA [17] and TTP [1] have reported. However, considering the studies on loudness change magnitude [18–21] that did report a greater bias for tones than for noise, our results would not be entirely surprising. Nevertheless, from the previous literature we would expect that, as the amount of information available decreased, the uncertainty of the task would have led to a greater anticipation of sounds’ passage, despite using white-noise as stimulus. Surprisingly, participants maintained the same level of accuracy across occlusion conditions, making it difficult to advocate for the explanatory hypothesis of an adaptive mechanism in these conditions. If participants were driven by safety, the greater uncertainty (i.e. the higher amount of occlusion) should have led to a greater tendency to anticipate the sound’s passage. Instead, participants maintained the same performance level, while changing their perceptual strategy. Specifically, the participants adopted a different preferential cue to estimate time-to-passage, from temporal (TTPt, TTPf) to spatial cues (fDist). As the amount of information provided by the stimuli was reduced by occlusion, theoretical and final TTP lost predictive strength, while the use of final distance increased the precision of the estimation of passage. These results indicate a change in the perceptual strategy as a function of the available information, even for final distances located on typical cones of confusion for sound localizations [25]. In other words, when the nature of the task implies more uncertainty, participants seem to rely more on positional variables than on temporal variables. In studies with the same procedure conducted in the visual modality, as in Mouta *et al*. (2012), anticipation was not found in the judgment of rigid motion stimuli, although a set of complex motion conditions was judged to pass sooner [7]. Also, these authors found a greater uncertainty for complex motion conditions, with both local and global information (SD ranging between 0.17 and 0.19s) in comparison with rigid motion (SD = 0.14s). In sum, with the visual modality participants seem to be more conservative in their estimates when the uncertainty of the task is higher. For the auditory modality we found that TTP continued to be predictive of subjects’ performance, despite the amount of occlusion and thus the level of uncertainty. Moreover, for auditory TTP, participants seem to rely on different variables depending on the amount of occlusion, and, interestingly, the same level of precision is maintained.

Additionally, we aimed to understand which psychoacoustic variables could contribute to the change in strategy of the perceptual cues. ITD_final_ and ILD_Loud_ as a function of the proportion of “later” responses revealed a possible insight into the computation of spatiotemporal cues from psychoacoustic variables. We found that the precision in estimating the time to passage of a sound increases for final distance as a function of the amount of occlusion, which is analogous to the tendency for a better quality of the fit for ITD_final_ as the amount of occlusion increases. Therefore, the final position of the stimuli might be perceptually extracted from this binaural cue. Additionally, the difference in ILD_Loud_ was more representative for the lower amount of occlusion, analogous to the predictive strength of theoretical Time to Passage in the same occlusion conditions. At least in this type of task, participants seem to accurately extract ILD_Loud_ in order to provide a temporal estimation of sounds’ passage. This finding provides further information to contextualize Neuhoff’s results on the judgement of loudness magnitude [18, 19] and spatial position judgment [19]. As Neuhoff (2001) also demonstrated, participants tend to perceive tones as stopping closer to them in comparison with white-noise. Although, in the present study, participants were not asked to judge distance, they seem to reliably use binaural information and final distance of the stimuli to be able to compute the time to passage.

It is noteworthy that, in the present study, we did not intend to simulate a sound source with ecological meaning. Our main purpose was to use a simple sound that could provide a baseline knowledge of looming sounds judgment, while presenting the cues of a sound from a real context—realistic sound–with binaural (ITD and ILD) preserved. The ecological perspective has been the main approach cited in the literature arguing in favour of the anticipatory tendency for the judgment of looming objects. It has been argued that the features of the stimuli used in Time-to-Arrival or Time-to-Contact experiments [17, 15] could contribute for a conservative performance. However, more studies are needed in order to further support the claim that the type of stimuli and/or its trajectory towards the observer contribute to the anticipatory results reported in the literature.

In our experiments, apart from the short presentation and response times we also have presented distances close to the listener. Specifically, the farthest distance was 1,29m from the participant, which is less than the distances used in other studies [1, 13, 33]. Hence, we may have two additional distinct aspects that might have influenced perceptual judgments. One concerning a space further from the observer, where participants’ seem to be more conservative in their perceptual estimates (e.g., Schiff & Detwiler, 1979 [15] with approaching squares), and another concerning the closest space in which participants’ seem to be more accurate (such as Mouta *et al*., [7]) with biological and complex motion). Additionally, an analogy can be made between the binaural cues in auditory perception and the depth cues in visual perception. In the visual modality, cues such as relative size, accommodation and vergence are most relevant for a near space up to 2 m [34, 35]. It has been shown that, in the auditory modality, we can rely more on interaural differences than solely loudness or frequency change, for distance judgments of static stimuli [36, 28]. Rosenblum *et al*. (1983) have demonstrated that intensity change is a robust cue, at least when the participant has to provide a temporal estimate of the sound source [1]. However, the trajectory of this stimulus was passing in front of the participant at 15m. Brungart, Durlach and Rabinowitz (1999) have addressed the study of localization of sound sources close to the participant, specifically with broadband stimuli [28]. Although not using dynamic sounds, these authors have reached the conclusion that ILD seems to be a more informative cue for proximal regions (up to 1 m from the listener’s head). Although our study supports this finding, it also indicates that ITD extracted from the end of stimulus’ presentation (ITD_final_) is informative when participant’s need to estimate the course of the stimulus. It is known that binaural differences are greater as the sound source approaches the interaural axis [36]. Therefore, it could be the case that monaural cues are more reliable for farthest distances, and binaural cues for distances closer to the participants’ head, at least for distances up to 1 m. However, this could be the case only for white-noise, as other studies using tones have shown that even with greater distances [37] participants are not accurate when relying on intensity.

Arguably, sound perception contributes to temporal perception in the same sense that vision contributes to spatial perception [38], which highlights our finding on the prevalence of final distance as the best predictor for auditory estimation to be quite surprising. Consequently, it is necessary to further explore this issue and to assess whether the usage of TTP information is task dependent and/or independent of the distance to the observer. It should also be noted that the methodology we used to address timing estimation–Time-to-Passage task—has been implemented in the visual modality with the same design. Occlusion periods are mandatory in these tasks, otherwise the estimation of the time of passage would be trivial, at least for a visual target. Moreover, a 2AFCT using an occlusion paradigm allowed to increase the uncertainty of the task in order to, on one hand analyse the effect of the task uncertainty on the accuracy of passage estimation of auditory looming stimuli; and on the other hand verify the role of different acoustic and psychoacoustic cues on TTP judgement varying the amount of information presented to the listener. Also, for the visual modality, it is known that when a moving object is occluded prior to contact with another object, observers tend to consistently underestimate the actual TTC [39]. In this work, the results revealed that the TTP estimates for white-noise looming stimuli were unaffected by an anticipatory perceptual bias, even for a 50% occlusion period.

In sum, we found that participants relied on different psychoacoustic cues provided by the stimulus, which enabled them to maintain the same level of performance. The shift in the perceptual strategy was consistent across participants and seems to be dependent on the amount of information that is available until the moment of the judgment.

## Conclusion

In conclusion, we presented a study on auditory Time-to-Passage estimation and analysed the contribution of physical and psychoacoustic cues extracted from the stimuli’s trajectory through space and time. The key finding of our study is that participants readily extract information from different cues provided by an auditory looming event. This is a novel result that brings greater insight to the study of looming events in a purely auditory task. Based on our results, we argue that no bias (i.e. anticipation) is observed in the estimation of time to passage of looming sounds, if the reliability of the information is maintained, even if provided by different cues. Also, the pattern of accuracy and precision in these tasks is maintained regardless the amount of occlusion. The perceptual strategy adopted by participants to maintain performance in the task, seems to be based on the usage of different informative cues. We hypothesize that final distance could be extracted from ITD_final_ (in higher amounts of occlusion) and that TTP could be extracted from ILD_Loud_ (for lower amounts of occlusion).

In the future we should also consider the role of the distance of the sound source from the participant in judging the time of passage. Specifically, studies should assess whether greater distances would influence performance and/or the perceptual strategies to estimate Time-to-Passage. Moreover, addressing these issues will help to better understand how timing estimates are achieved in a wider range of situations. All in all, this work contributed to the discussion around the anticipation of looming sounds by trying to identify the cues in which participants rely the most to compute Time-to-Passage.

## Supporting information

S1 Dataset Pooled dataset of study with the variables used for analysis. (CSV)

S1 Fig Schematic representation of two stimuli in the 3 occlusion conditions. The figure represents two examples of a stimuli in all occlusion conditions. The stimulus corresponding to a Time-to-Passage of 0,59 s is represented on the left panel. It starts approaching the listener at an initial distance of 0,78 m and at a constant speed of 1,32 m/s. However, due to different occlusion periods (represented by different colours), the final position at which the stimulus is audible varies. The last position where the stimulus is audible before occlusion is at 0,12 m before de participant’s ear plane (for 50% occlusion), at 0,14 m after participant’s ear plane (for 30% occlusion) and, at 0,41 m after participant’s ear plane (for 10% occlusion). The dashed lines represent the trajectory of the stimulus during the occlusion period, until the moment of judgment. The stimulus corresponding to a Time-to-Passage of 1,697 s is represented on the right panel. It starts approaching the listener at an initial distance of 1,29 m and at a constant speed of 0,76 m/s. The last position where the stimulus is audible before occlusion is at 0,91 m before de participant’s ear plane (for 50% occlusion), at 0,76 m before participant’s ear plane (for 30% occlusion) and, at 0,61 m before participant’s ear plane (for 10% occlusion).
(TIF)

S1 Multimedia Audio files of the two stimuli represented in w in all occlusion conditions. (ZIP)

S1 Table Range of values of the variables plotted for analysis. Due to different presentation times, the variables assume different distribution ranges and therefore, different Points of Objective Simultaneity (POS) as s function of the occlusion period.
(TIF)
