# Supplementary figures and images for: Judging time-to-passage of looming sounds: Evidence for the use of distance-based information
Source: PLoS One. 2017 May 22;12(5):e0177734. doi: 10.1371/journal.pone.0177734 (PMC5439697; doi:10.1371/journal.pone.0177734)

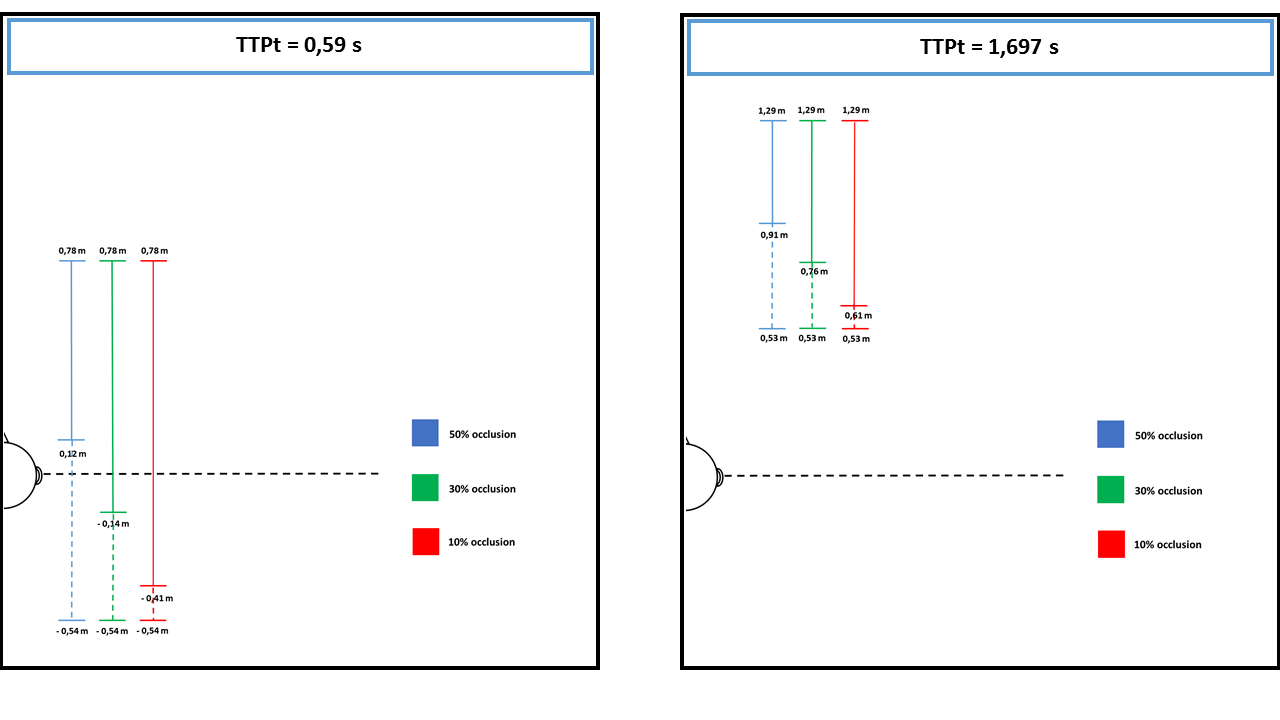

Supplement: S1 Fig — The figure represents two examples of a stimuli in all occlusion conditions. The stimulus corresponding to a Time-to-Passage of 0,59 s is represented on the left panel. It starts approaching the listener at an initial distance of 0,78 m and at a constant speed of 1,32 m/s. However, due to different occlusion periods (represented by different colours), the final position at which the stimulus is audible varies. The last position where the stimulus is audible before occlusion is at 0,12 m before de participant’s ear plane (for 50% occlusion), at 0,14 m after participant’s ear plane (for 30% occlusion) and, at 0,41 m after participant’s ear plane (for 10% occlusion). The dashed lines represent the trajectory of the stimulus during the occlusion period, until the moment of judgment. The stimulus corresponding to a Time-to-Passage of 1,697 s is represented on the right panel. It starts approaching the listener at an initial distance of 1,29 m and at a constant speed of 0,76 m/s. The last position where the stimulus is audible before occlusion is at 0,91 m before de participant’s ear plane (for 50% occlusion), at 0,76 m before participant’s ear plane (for 30% occlusion) and, at 0,61 m before participant’s ear plane (for 10% occlusion). (TIF) [file pone.0177734.s002.tif]

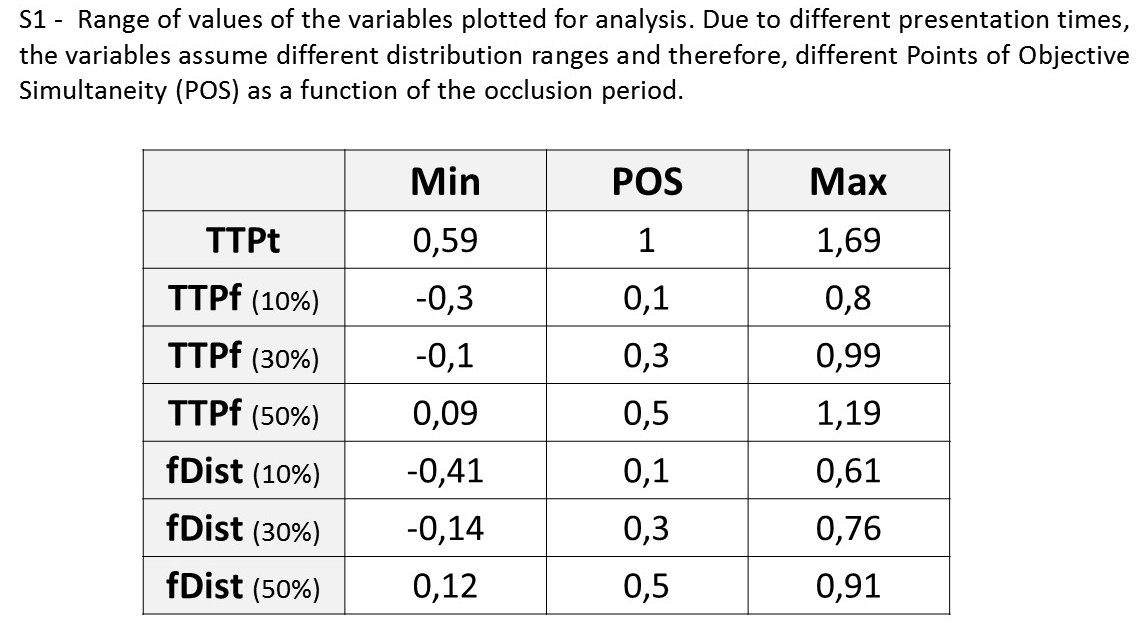

Supplement: S1 Table — Due to different presentation times, the variables assume different distribution ranges and therefore, different Points of Objective Simultaneity (POS) as s function of the occlusion period. (TIF) [file pone.0177734.s004.tif]
